# Characteristics of Health Systems Operating Medicare Advantage Plans

**DOI:** 10.1001/jamahealthforum.2024.3536

**Published:** 2024-11-08

**Authors:** Aaron Hedquist, Eric Yu, Pasha Hamed, E. John Orav, Austin Frakt, Thomas C. Tsai

**Affiliations:** 1 Department of Health Policy and Management, Harvard T.H. Chan School of Public Health, Boston, Massachusetts; 2 Department of Medicine, Brigham & Women’s Hospital, Boston, Massachusetts; 3 Partnered Evidence-Based Policy Resource Center, Boston VA Healthcare System, Boston, Massachusetts; 4 Department of Health Law, Policy and Management, Boston University School of Public Health, Boston, Massachusetts; 5 Department of Surgery, Brigham and Women’s Hospital, Boston, Massachusetts

## Abstract

This cross-sectional study describes Medicare Advantage plans owned or operated by health care systems in the US.

## Introduction

Health care delivery has rapidly transitioned from independent physicians and hospitals to integrated delivery networks. More than three-quarters of inpatient facilities are affiliated with a health system.^1,2^ With increasing financial and provider organization integration under value-based care contracts, many systems have elected to function as both payer and provider organization by owning and operating health insurance plans, including Medicare Advantage (MA). This may be motivated by health systems responding to decreased reimbursement or claims denials from MA health plans owned by insurance companies; however, concerns that health systems may be alternatively motivated to offer narrow networks of only practitioners affiliated with their system are increasing. Contemporaneous data on the trends and characteristics of health systems owning or operating MA health insurance plans are needed.

## Methods

In this study, we used the Agency for Healthcare Research and Quality (AHRQ) Compendium of US Health Systems to identify health systems operating MA plans through direct ownership or partial management with another organization. We followed the STROBE reporting guideline. The study was exempt from IRB review, and informed consent was waived because data were deidentified.

The compendium includes information from 2018 to 2022. We linked these data to the AHRQ Compendium Hospital Linkage file and the American Hospital Association Annual Survey database to inform facility-level characteristics. Contract-level MA plan characteristics were derived from the MA Plan Directory. Patient characteristics and enrollment data were calculated from the 100% inpatient sample of traditional Medicare claims data from 2021 and the beneficiary summary file. Using the Private Equity (PE) Stakeholder Project’s PE Hospital Tracker, we identified hospitals with PE investment during the study period.^3^

We report the number of health systems operating MA plans and longitudinal trends in the number of beneficiaries enrolled. We then performed *t* and χ^2^ tests to compare the ownership and structural features of health systems that self-reported offering vs not offering MA plans in 2022. Statistical significance was determined with a 2-sided *P* < .05. Data were analyzed with SAS 9.4 (SAS Institute).

## Results

In 2022, the compendium identified 640 health systems; 121 (18.9%) self-reported operating an affiliated MA plan. The number of beneficiaries enrolled in system-operated MA plans increased from 3 313 709 to 4 173 688 between 2018 and 2022 (Table 1). In 2022, this represented 13.1% of all MA beneficiaries. The number of identified system-operated MA plans also steadily increased from 135 to 196 from 2018 to 2022.

**Table 1.** Top 20 Health Systems by 2022 System-Operated Medicare Advantage (MA) Plan Enrollment

Health system	States with significant enrollment^a^	MA contracts, No.^b^	MA enrollment, No.^c^		Change (2018-2022), %
			2018	2022	
Kaiser Permanente	AZ, CA, CO, DC, GA, HI, ID, IL, MD, NV, OR, SD, TX, VA, WA	7	1 624 496	1 841 675	13.40
Allegheny Health Network	AL, AR, AZ, CA, CO, CT, DE, FL, GA, IA, ID, IL, IN, KS, KY, LA, MA, MD, MI, MO, NC, NE, NH, NJ, NM, NV, NY, OH, OR, PA, SC, TN, TX, VA, VT, WA, WI, WV	7	315 859	377 126	19.40
Corewell Health	AZ, FL, MI, NV, OH	4	145 450	227 137	56.20
UPMC	OH, PA	5	182 859	201 808	10.40
Fairview Health Services	MN, WI	5	NA	140 094	NA
Ascension Health	AL, FL, IL, IN, KS, MI, NY, OK, TN, TX, WI	23	69 808	122 617	75.60
Geisinger	PA	4	96 160	96 659	0.50
Henry Ford Health	MI	4	67 133	87 278	30.00
Froedtert and the Medical College of Wisconsin	WI	3	65 761	71 577	8.80
Providence	OR, WA	3	60 068	65 337	8.80
Trinity Health	AL, DE, FL, IA, ID, IN, MA, MD, MI, NC, NJ, NY, OH, PA, TX	20	110 559	63 347	−42.70
Marshfield Clinic Health System	WI	2	50 736	63 234	24.60
Presbyterian Healthcare Services	NM	2	45 130	55 928	23.90
HealthPartners	IA, IL, MN, ND, SD, WI	4	67 381	54 311	−19.40
The Carle Foundation	IA, IL, IN, NC, OH, WA	6	29 394	47 347	61.10
Intermountain Healthcare	ID, NV, UT	1	41 311	44 095	6.70
Unitypoint Health	IA, IL, MN, WI	4	18 120	38 845	114.40
Health First	FL	1	38 087	34 987	−8.10
Saint Francis Health System	OK	2	3255	31 001	852.40
Gundersen Health System	IA, IL, MN, WI	2	16 776	30 808	83.60
All others	34	87	265 366	478 477	80.31
All combined	44 + DC	196	3 313 709	4 173 688	25.95

Abbreviations: NA, not applicable; UPMC, University of Pittsburgh Medical Center.

^a^ Significant enrollment was calculated using the MA Enrollment by State/County/Contract file. Only states that had 11 or more beneficiaries enrolled in a single county were listed. Two states (Maine and Rhode Island) had adequately large MA contracts that were unaffiliated with health systems. The remaining 4 states (Arkansas, Mississippi, Montana, and Wyoming) did not include any MA contracts with significant enrollment.

^b^ Information on health system–operated MA contracts is extracted from the Compendium of US Health Systems. The compendium identified MA insurance contracts for 76 systems by matching parent organizations of health systems with MA contracts available in the Centers for Medicare & Medicaid Services MA Plan Directory.

^c^ The compendium aggregates enrollment information published in the Centers for Medicare & Medicaid Services MA Plan Directory.

Health systems operating MA plans were on average larger (2177 vs 780 beds; *P* < .001; Table 2) with more clinicians (2515 vs 744 physicians; *P* < .001) and affiliated nursing homes (5.14 vs 0.89 nursing homes; *P* = .02) than systems not operating MA plans. Systems treating lower proportions of White Medicare beneficiaries (73.7% vs 78.8%; *P* = .02) were associated with operating MA plans.

**Table 2.** Characteristics of Health Systems Self-Reporting Operating Medicare Advantage (MA) Plans in 2022

Characteristic^a^	Systems operating MA plans (n = 121)	Systems not operating MA plans (n = 411)	*P* value^b^
Ownership status, %^c^			
Nonprofit	68.4	69.4	<.001
Church affiliated	5.6	11.6	
Public/government, nonfederal	22.9	17.4	
For profit/investor owned	3.2	1.7	
Size, average No.			
Beds	2177	780	<.001
Physicians	2515	744	<.001
Nursing homes	5.14	0.89	.02
Discharges	101 279	35 519	<.001
Profitability and market			
Average net revenue per discharge, $	45 504	52 226	.30
Average systemwide high uncompensated care, %	19.0	18.6	.87
Includes any PE-backed hospitals, %^d^	5.8	2.4	.07
Average Herfindahl-Hirschman Index^e^	0.12	0.14	.21
Region, %^f^			
Midwest	30.6	25.6	.38
Northeast	17.4	22.1	
South	29.8	33.8	
West	22.3	18.5	
No. of states reached, %			
1	70.2	84.9	<.001
2	13.2	9.5	
≥3	16.5	5.6	
Systemwide teaching intensity, %^g^			
Nonteaching	11.6	31.4	<.001
Minor teaching	52.1	40.7	
Major teaching	36.4	27.9	
Proportion of discharges by patient characteristic, %			
Medicaid^e^	24.0	23.7	.78
Medicare^e^	43.4	44.9	.28
Dual eligible^e^	27.1	28.1	.63
Asian^h^	2.8	1.7	.04
Black^h^	12.0	9.7	.07
Hispanic^h^	8.3	6.3	.09
Native Hawaiian or Pacific Islander^h^	0.8	0.9	.83
White^h^	73.7	78.8	.02

Abbreviation: PE, private equity

^a^ Information on health systems is extracted from the Compendium of US Health Systems. The compendium identifies plan offerings by health systems via the self-reported information available in the 2021 American Hospital Association Annual Survey (the latest survey available when the compendium was published). The 2022 compendium is missing survey responses from 108 systems.

^b^ Unadjusted *P* values are shown, but to accommodate multiple comparisons, results that remain significant after a Benjamini-Hochberg multiple testing procedure with a false discovery rate of 5% have been highlighted.

^c^ Health systems can be comprised of hospitals with different ownership types. The compendium determines the predominate systemwide status by calculating the number of beds associated with each ownership type.

^d^ Hospitals with PE investments were identified using the PE Stakeholder Project’s PE Hospital Tracker.

^e^ These variables were calculated using a discharge-weighted average of affiliated facilities.

^f^ The compendium aggregates health systems to their highest levels of ownership, so a primary state is chosen for a system spanning multiple states.

^g^ The compendium calculates systemwide teaching intensity using the systems’ resident-to-bed ratio of affiliated nonfederal general acute care hospitals. None, minor, and major thresholds are set at ratios of 0, less than 0.25 (but greater than 0), and equal to or greater than 0.25, respectively.

^h^ The racial and ethnic composition of each system was calculated using 2021 Traditional Medicare Inpatient Claims from affiliated general acute care hospitals.

Compared with other ownership types, a disproportionate number of church-affiliated health systems operated MA plans. Further, health systems operating in 3 or more states were more likely to offer MA plans than more concentrated systems. System-operated MA plans had higher overall quality star ratings (4.38 vs 4.03; *P* < .001) and patient satisfaction (4.41 vs 3.78; *P* < .001) compared to plans not operated by health systems.

## Discussion

Nearly 1 in 7 MA beneficiaries are enrolled in system-operated MA plans, which remain a consistent source of Medicare enrollment. The findings of this study suggest that larger and church-affiliated health systems are associated with a higher likelihood of operating an MA plan. System-operated MA plans were associated with higher quality ratings and patient satisfaction than unaffiliated MA plans. This aligns with prior research suggesting system-operated plans may be more cost-effective, efficient, and of higher quality.^4^ Limitations included that this analysis could not determine causation or why certain factors are associated with system-operated MA plans. Further research is warranted on whether health system–operated MA plans provide better value for Medicare beneficiaries through aligned incentives with clinicians.

## Notes

Supplement 1. eMethods

Supplement 2. Data Sharing Statement
