# Supplementary material for: Characteristics of Health Systems Operating Medicare Advantage Plans
Source: JAMA Health Forum. 2024 Nov 8;5(11):e243536. doi: 10.1001/jamahealthforum.2024.3536 (PMC11549652; doi:10.1001/jamahealthforum.2024.3536)
Supplement: Supplement 1. — eMethods [file jamahealthforum-e243536-s001.pdf]

Supplemental Online Content

Hedquist A, Yu E, Hamed P, Orav EJ, Frakt A, Tsai TC. Characteristics of health systems operating Medicare Advantage plans. *JAMA Health Forum*. 2024;5(11):e243536. doi:10.1001/jamahealthforum.2024.3536

eMethods. Data Sources and Linkages

This supplemental material has been provided by the authors to give readers additional information about their work.

© 2024 Hedquist A et al. *JAMA Health Forum.*

eMethods. Data Sources and Linkages

Data Source	Notes	Linkage
**Agency for Healthcare Research and Quality, Compendium of U.S. Health Systems (2018 – 2022)**	The Compendium of U.S. Health Systems, developed by AHRQ, combines information from IQVIA One Key and the American Hospital Association (AHA) Annual Survey to identify Health Systems. In their technical documentation, AHRQ defines a health system as an organization with "at least one hospital and at least one group of physicians that provides comprehensive care (including primary and specialty care) who are connected with each other and with the hospital through common ownership or joint management." Functionally, the compendium requires hospitals to own or manage at least one non-federal general acute care hospital, fifty or more physicians, and ten primary care physicians. During the study period, the number of health systems identified by the compendium remained largely consistent from 637 in 2018 to 640 in 2022.<br><br>The compendium includes characteristic information derived from IQVIA One Key, AHA Annual Survey, and the Centers for Medicare and Medicaid Healthcare Provider Cost Reporting Information System (HCRIS). In this study, we use the following information:<br><br>1) Health System Profit Status<br>2) Teaching Status<br>3) Operational Reach<br>4) Number of MDs<br>5) Number of Beds<br>6) Number of Nursing Homes<br>7) Number of Discharges<br>8) Net Revenue per Discharge<br>9) High Uncompensated Care Burden<br>10) Self-Reported Medicare Advantage Plan<br>11) AHRQ Identified Contracts and Enrollment	System ID<br><br>MA Contract IDs
**Agency for Healthcare Research and Quality, Compendium of U.S. Health Systems, Hospital Linkage File (2018 – 2022)**	Additionally, AHRQ publishes a linkage file that includes facility level information compiled from the IQVIA One Key, AHA Survey, and HCRIS. This data and technical documentation are publicly available via the AHRQ website.<br><br>In this study, we use the Linkage File to determine the list the hospitals affiliated with the health systems listed in the Compendium. We use the hospitals' facility IDs (CCNs) to access relevant Medicare claims data (see below).	System ID<br><br>Hospital Facility ID (CCN)<br><br>AHA ID

© 2024 Hedquist A et al. *JAMA Health Forum.*

**American Hospital Association Annual Survey (2021)**	In addition to the publicly available information provided by AHRQ, we supplement our analysis with information from the AHA Annual Survey, including the proportion Medicaid and Medicare Discharges.	AHA ID
**Private Equity Stakeholder Projects, Private Equity Hospital Tracker (Cumulative)**	To identify hospitals with any private equity investment, we used the Private Equity Hospital Tracker. This database tracks private equity investments announced in news searches, available in Pitchbook, and documented in CMS databases. We linked information in this database to information in the AHRQ Hospital Linkage File via the Hospital Facility ID (CCN).	Hospital Facility ID (CCN)
**Traditional Medicare Inpatient Claims and Master Beneficiary Summary File**	We used traditional Medicare inpatient claims combined with the Master Beneficiary Summary File to calculate the proportion of discharges within each system by patient demographics. This included dual-eligibility status and race and ethnicity characteristics.	Hospital Facility ID (CCN)
**CMS Medicare Advantage Plan Directory**	The CMS Plan Directory includes information on MA contract-level enrollment and star ratings. We use this dataset to compare the quality of plans affiliated and unaffiliated with health systems.	MA Contract IDs

© 2024 Hedquist A et al. *JAMA Health Forum.*
