# Supplementary material for: Characteristics of Health Systems Operating Medicare Advantage Plans
Source: JAMA Health Forum. 2024 Nov 8;5(11):e243536. doi: 10.1001/jamahealthforum.2024.3536 (PMC11549652; doi:10.1001/jamahealthforum.2024.3536)
Supplement: Supplement 2. — Data Sharing Statement [file jamahealthforum-e243536-s002.pdf]

Data Sharing Statement

Hedquist. Characteristics of Health Systems Operating Medicare Advantage Plans. *JAMA Health Forum*. Published November 08, 2024. doi:10.1001/jamahealthforum.2024.3536

Data
Data available: No

Additional Information
Explanation for why data not available: Unable to share data due to data use agreement
